# Integrative analysis identifies LHFPL6 as a CAF-specific prognostic biomarker in colorectal cancer

**DOI:** 10.1007/s10238-025-01954-y

**Published:** 2025-11-18

**Authors:** Xiaoyong Zheng, Yan Bai, Jianping Sun, Yage Yang, Xuefeng Lv, Xinyu Zhang, Mengyao Shi, Jinhua Zhao, Dawei Shi, Zhuoran Chen, Jing Wang, Hui Yee Yeo, Yajie Ma, Sitian He, Mengle Peng

**Affiliations:** 1 Department of Gastroenterology, The Third People’s Hospital of Henan Province, Zhengzhou, 450006 Henan China; 2 Department of Gastroenterology, Zhengzhou First People’s Hospital, Zhengzhou, 450000 Henan China; 3 Department of Pathology, Zhengzhou Ninth People’s Hospital, Zhengzhou, 450000 Henan China; 4 Department of Clinical Laboratory, The Third Affiliated Hospital of Zhengzhou University, Zhengzhou, 450052 Henan China; 5 Department of Medical Affair, The Third People’s Hospital of Henan Province, Zhengzhou, 450006 Henan China; 6 School of Medicine, Duke University, Durham, 27705 USA; 7 Liggins Institute, University of Auckland, Auckland, 1023 New Zealand; 8 Department of Clinical Laboratory, Hainan General Hospital (Hainan Affiliated Hospital of Hainan Medical University, Hainan Medical University, Haikou, 571199 China; 9 Department of Clinical Laboratory, The Third People’s Hospital of Henan Province, Zhengzhou, 450006 Henan China

**Keywords:** Colorectal cancer, Cancer-associated fibroblasts, LHFPL6, Prognosis, Biomarker

## Abstract

Recent studies have emphasized the contributions of cancer-associated fibroblasts (CAFs) to cancer initiation, immune suppression, and treatment resistance. Advancing our molecular understanding of CAFs is therefore crucial to improve outcomes for colorectal cancer (CRC) patients. In this study, integrative analysis of single-cell RNA-sequencing data from GSE166555 and GSE144735 identified ten cellular subpopulations. Consensus clustering exhibited four distinct CAF subtypes, with the C4 subtype showing elevated expression of most CAF-specific genes, and predominant enrichment of stromal activation signatures. Functional enrichment analysis of C4-specific transcripts showed dominant enrichment in extracellular matrix (ECM) remodeling pathways. Univariate Cox regression analysis identified eight survival-critical genes, among which LHFPL6 demonstrated the highest specificity and sensitivity for discriminating C4 (AUC = 0.98). Multiple cohorts further confirmed that higher LHFPL6 expression was associated with worse survival outcomes across diverse clinical endpoints (all *p* < 0.05). Multivariate Cox regression analysis established LHFPL6 as an independent prognostic factor (HR = 5.889, *p* = 0.017). Additionally, LHFPL6 was predominantly expressed in fibroblasts and positively correlated with CAF scores. Co-expressed genes of LHFPL6 showed significant enrichment in ECM signatures and epithelial–mesenchymal transition (EMT) pathways. Functional experiments using CAF-tumor co-culture models demonstrated that LHFPL6 knockdown suppressed SW480 malignant behaviors, as evidenced by reduced proliferation, migration, and invasion. This study demonstrates that LHFPL6 serves as a promising prognostic biomarker and potential therapeutic target for CRC.

**Supplementary Information:**

The online version contains supplementary material available at 10.1007/s10238-025-01954-y.

## Introduction

Colorectal cancer (CRC) accounted for approximately 1.9 million newly diagnosed cancer cases and 900,000 related fatalities estimated in 2022 and remains one of the most prevalent malignancies globally [1]. The current therapeutic approaches for CRC include surgery, chemotherapy, and radiotherapy, which have notably enhanced patient survival rates [2]. However, many challenges, including drug resistance, metastasis, recurrence, and aggressive tumor behavior, limit the accessibility and effectiveness of systemic anticancer therapy [3]. Thus, achieving early diagnosis and developing more efficient, precise therapies with reduced toxicity have become critical goals in CRC management.

Immunotherapy, comprising immune checkpoint inhibitors (ICIs) and adoptive cellular therapeutic agents (ACTs), has become a well-established therapeutic modality for CRC [4]. The efficacy of immunotherapy varies considerably based on individual heterogeneity, tumor type, and the complexity of the tumor microenvironment (TME) [5]. The TME, consisting of tumor cells, stromal and immune cells, and diverse secreted factors, is critically involved in tumor formation and progression, modulating tumor behavior, as well as targeted therapy response [6–8]. Within this dynamic environment, stromal cells, including cancer‐associated fibroblasts (CAFs), interact with tumor cells as the tumor grows, leading to a mutual influence that forms the basis for TME development [9, 10]. CAFs are derived from multiple cellular sources, such as resident fibroblasts, bone marrow-derived mesenchymal stem cells, tumor cells, and endothelial cells [11]. Through mechanisms including extracellular matrix (ECM) remodeling, cell–cell interactions (mediated by intercellular adhesion), promotion of immune evasion, CAFs exert significant effects on the TME [12, 13]. Additionally, CAFs can facilitate tumor immune evasion through the secretion of diverse soluble mediators, comprising proteins, cytokines, and chemokines, which are major contributors to resistance against ICI therapy [14]. Despite their functional importance, CAFs display significant heterogeneity, as evidenced by diverse subpopulation markers. Several markers have been employed to label CAFs, such as alpha smooth muscle actin (α-SMA), fibroblast activation protein (FAP), platelet-derived growth factor receptor (PDGFR), and podoplanin (PDPN) [15–17]. Targeting established biomarkers of CAFs can inhibit tumor growth and metastasis while enhancing the efficacy of existing therapies [18]. The heterogeneity of CAFs results in discordant biomarker expression across different tumor types and individuals, thereby impeding the uniform identification and application of these markers [19]. This underscores the need to investigate potential CAF-specific genes, which may provide targeted therapeutic strategies for CRC patients.

The integration of single-cell RNA sequencing (scRNA-seq) with bulk RNA-seq represents a state-of-the-art bioinformatic strategy that enables the identification of previously uncharacterized CAF subpopulations and novel molecular biomarkers. In this study, we used scRNA-seq data in CRC to finely cluster cell types in the TME and identified CAF subtypes based on CAF-specific genes. We systematically evaluated stromal infiltration levels of CAF subtypes and found that the C4 subtype was closely related to the CAF characteristics represented by CAF-specific genes. The C4 subtype was dominantly enriched in ECM remodeling pathways. In addition, we identified a prognostic CAF-specific gene, LHFPL6, and confirmed that LHFPL6 knockdown attenuated SW480 malignancy, suppressing proliferation, migration, and invasion using CAF-tumor co-culture models. This study enhances our understanding of CAF-specific subpopulations and genes associated with CRC progression and prognosis and provides insights into the roles of LHFPL6 in cancer development.

## Materials and methods

### Data collection

ScRNA-seq data were obtained from the GSE166555 and GSE144735 datasets and analyzed utilizing the R package Seurat. The raw gene expression matrix from 10 × Genomics was initially loaded and processed to construct a Seurat object. Quality control filtration was implemented to exclude low-quality cells based on established criteria: cells retaining 200–5,000 detected genes, 500–40,000 UMI counts, mitochondrial gene content < 20%, and log10 (nFeature/nCount) ratio > 0.8. Doublet detection and removal were conducted using the Scrublet method with an expected doublet rate parameter set at 5%. Two Gene Expression Omnibus (GEO) datasets (GSE87211, GSE71187) and CRC genomic data from The Cancer Genome Atlas (TCGA) program were obtained as independent validation cohorts.

### Preprocessing of scRNA-seq data

Subsequent processing included data normalization via log transformation, identification of 2000 highly variable genes (HVGs) for feature selection, and batch effect correction using Harmony integration. Dimensionality reduction was achieved through principal component analysis (PCA) followed by nonlinear visualization techniques (UMAP). Cell clustering optimization was performed by testing resolution parameters ranging from 0.05 to 4.

### Annotation of cellular subpopulations

Cell population annotation employed two complementary approaches: canonical marker gene identification and COSG algorithm-based classification. Cell-type annotation was performed according to expression patterns of specific marker genes, including B cells (CD79A, CD79B and MS4A1), plasma cells (TNFRSF17, MZB1 and IGHA1), T/NK cells (CD2, CD3D, CD3E, TRAC and TRBC1), myeloid cells (CD14, CD68, CD163, S100A8 and FCGR3A), dendritic cells (ITGAX, CD1C and FCER1A), and mast cells (TPSAB1 and MS4A2). Additionally, nonimmune cell types were characterized, including epithelial cells (EPCAM, CD24, KRT18 and KRT8), endothelial cells (PECAM1, CDH5, ENG, CLDN5 and VWF), pericyte cells (MYH11, ACTA2, NDUFA4L2, COX4I2, RGS5 and NOTCH3), and fibroblast cells (COL1A1, COL1A2 and COL3A1).

### Differential gene analysis

Cluster-specific differentially expressed genes (DEGs) were computationally identified using FindAllMarkers or FindMarkers functions, enabling systematic comparison of transcriptional profiles between tumor tissues and normal tissues. For a gene to be considered differentially expressed, it must be detected in at least 25% of cells and exhibit a minimum log-fold change of 0.25. Statistical significance of identified DEGs was determined using the Wilcoxon rank-sum test. An adjusted p-value (false discovery rate, FDR) < 0.05 was required to establish significant differential expression. To assign cellular lineages to specific clusters, we performed systematic comparisons utilizing cluster-specific marker genes and established lineage-defining markers. Finally, we primarily focus on fibroblast cells and identified CAF-specific genes. These findings were visualized using FeaturePlot.

### Consensus clustering

To delineate potential CAF subpopulations, we performed consensus clustering on scRNA-seq data focusing on CAF-specific genes. The expression matrix was constructed by extracting transcript counts corresponding to CAF-specific genes, with rows denoting genes and columns representing individual single-cell profiles. Data normalization was achieved through Z-score transformation, standardizing each gene's expression values to a mean of 0 and a standard deviation of 1, thereby mitigating batch effects and scaling disparities across heterogeneous gene expression ranges. Consensus clustering was implemented via the consensusClusterPlus R package. Key parameters were configured as follows: (1) K range: Candidate cluster numbers (K) were evaluated from 2 to 8; (2) Resampling: A subsampling proportion (pItem) of 0.8 was applied to ensure robustness against overfitting; (3) Replicates: 1,000 iterations were executed to stabilize consensus indices; (4) Clustering algorithm: The Partitioning Around Medoids (PAM) algorithm was selected due to its enhanced tolerance to outliers compared to centroid-based methods (e.g., k-means). PAM optimizes clustering by minimizing the sum of dissimilarities between each cell and its assigned cluster medoid, a representative member robust to noise. This integrative framework leverages ensemble learning principles to quantify cluster stability, enabling data-driven determination of optimal K while accounting for stochastic variability inherent in high-dimensional single-cell datasets.

### Assessment of stromal infiltration

The ESTIMATE (Estimation of STromal and Immune cells in MAlignant Tumors using Expression data) algorithm provides a robust bioinformatics framework for systematically evaluating stromal infiltration levels (Stromal Score) within the TME. This method employs a curated gene set encompassing stromal cell-specific markers (e.g., fibroblasts, endothelial cells) and ECM remodeling-associated genes (StromalSignature genes). By normalizing the gene expression matrix, the algorithm quantifies pathway activity associated with stromal biological processes, generating a continuous Stromal Score that exhibits stromal component abundance in tumor tissues.

### Gene set enrichment analysis

To elucidate the biological pathways enriched in the fibroblast C4 subpopulation, we performed GSEA utilizing the R package clusterProfiler. Subsequent functional annotation included: 1) Gene Ontology (GO) enrichment analysis; 2) Kyoto Encyclopedia of Genes and Genomes (KEGG) pathway analysis; and 3) Hallmark gene set analysis from the Molecular Signatures Database (MSigDB). The criterion for statistical significance was FDR < 0.05.

### Univariate cox regression analysis

Univariable Cox regression was conducted to assess the prognostic value of CAF-specific genes in CRC. Genes demonstrating significant associations with survival outcomes were characterized by corresponding hazard ratios (HRs), 95% confidence intervals (CIs), and p-values. Genes with HR > 1 indicate increased risk (poor prognosis); genes with HR < 1 suggest protective effects.

### Multivariate cox regression analysis

To determine whether LHFPL6 is an independent prognostic factor, we performed multivariate Cox regression analysis. LHFPL6 expression along with clinically relevant variables (age, gender, and tumor stage) was included in the model. HRs with 95% CIs were calculated, and the proportional hazards assumption was assessed using Schoenfeld residual tests.

### Survival analysis and ROC curves

To identify the prognostic impact of candidate genes identified by univariate Cox regression, Kaplan–Meier survival curves were generated using the "survminer" package in R. Patients were stratified into high and low expression groups based on median expression values. Survival differences were assessed using log-rank tests. For time-dependent prediction assessment, ROC curves with the area under the curve (AUC) calculations were generated using the "timeROC" R package.

### Co-expression network analysis

To investigate the functional implications of LHFPL6-associated genes in CRC, we performed co-expression network analysis using the TCGA-CRC cohort. The top 50 co-expressed genes of LHFPL6 were identified based on Pearson correlation coefficients. Statistical significance was FDR < 0.05.

### Primary CAF isolation

Primary CRC specimens were acquired from patients during therapeutic surgical resection procedures at the Third People’s Hospital of Henan Province (Ethics Approval No. 2024SZSYLCYJ1101). Tissue processing was performed under sterile conditions as follows: Fresh tumor specimens were sequentially rinsed in 5 × trypsin-supplemented PBS (Servicebio, Wuhan) with gentle agitation to eliminate blood contaminants and necrotic debris. After meticulous removal of adipose tissue using ophthalmic scissors, the specimens were minced into 1-mm^3^ fragments using crossed scalpels. Enzymatic dissociation was conducted with 1 mg/ml collagenase IV (Thermo Fisher Scientific, Shanghai) in a shaking water bath (37 °C, 120 rpm) for 2 h. The resulting cell suspension was sequentially filtered through 200-μm nylon meshes, centrifuged at 300 × g for 5 min, and resuspended in high-glucose DMEM (Gibco; Thermo Fisher Scientific) supplemented with 10% heat-inactivated FBS (Gibco) and 1% penicillin/streptomycin. Primary cultures were established in 60-mm collagen-coated dishes (Corning) maintained at 37 °C with 5% CO₂. Differential adhesion was achieved through medium replacement after 72 h to selectively enrich CAFs.

### Cell culture, transfection, and co-culture

The human CRC cell line SW480 was maintained in high-glucose DMEM (Gibco, USA) supplemented with 10% heat-inactivated fetal bovine serum (FBS; Thermo Fisher Scientific) and 1% penicillin/streptomycin (100 U/mL penicillin, 100 μg/mL streptomycin; Sigma-Aldrich), under standard culture conditions (37 °C, 5% CO₂, humidified atmosphere).

For LHFPL6 knockdown in CAFs, siRNA duplexes targeting human LHFPL6 and scrambled negative control siRNA (Tsingke Biotechnology, Beijing, China) were designed using BLOCK-iT™ RNAi Designer (Thermo Fisher). Reverse transfection was performed using jetPRIME® transfection reagent (Polyplus-transfection®, Illkirch, France) at a 2:1 (v/w) reagent-to-siRNA ratio when CAFs reached ~ 50% confluence. At 48 h post-transfection, CAFs were trypsinized (0.25% Trypsin–EDTA; Gibco), washed with PBS, and co-cultured with SW480 cells in Transwell® inserts (0.4-μm pore; Corning) at a 1:5 effector-to-target ratio. SW480 cells were harvested 48 h post-co-culture for subsequent functional analyses.

### Cell proliferation assay

Cell Counting Kit-8 (CCK-8) assay (Dojindo, Tokyo, Japan) was utilized to assess the rate of cell proliferation according to the manufacturer’s instructions. At indicated time points (0, 24, 48, 72, and 96 h), 10 μL of CCK-8 solution was added to each well and incubated for 2 h at 37 °C. A microplate reader (ELX808, BioTek, USA) was used to measure the absorbance at 450 nm. Finally, we calculated the cell proliferation rate according to the control group. Cell proliferation rates were calculated relative to the control group and expressed as mean ± standard deviation (SD) from three independent experiments.

### EdU

EdU (5-ethynyl-2′-deoxyuridine) is a thymidine analog widely used to label proliferating cells in S-phase. Cells were incubated with EdU (10 μM, 2 h) to allow its incorporation into newly synthesized DNA. After washing with PBS, cells were fixed with 4% paraformaldehyde and permeabilized with 0.5% Triton X-100. A copper(I)-catalyzed click reaction was performed using a fluorescent azide probe (BeyoClick™ EdU-594) to covalently conjugate the EdU-incorporated DNA, enabling visualization under fluorescence microscopy. Cell nuclei were counterstained with Hoechst 33,342. For quantification, the EdU-positive cell ratio was calculated as (EdU + cells / total cells) × 100% using imaging software (ImageJ).

### Migration and Invasion assay

The Transwell assay evaluates cell migration or invasion using chamber inserts with porous membranes (8-μm pores) (Corning, USA). For invasion assays, the upper was coated with Matrigel (diluted in serum-free medium); migration assays omitted this step. Serum-starved cells (5 × 10^4^ cells/well) were plated in the upper chamber with serum-free medium, while the lower chamber contained 10% FBS medium. After 40 h, non-migrated/invaded cells were removed from the upper chamber using cotton swabs. Migrated/invaded cells on the lower membrane surface were fixed with 4% paraformaldehyde, stained with 0.1% crystal violet, and imaged by microscope. Quantification was performed by counting cells in ≥ 3 random fields per insert using microscopy and ImageJ software.

### Wound healing

The scratch assay evaluates cell migration by creating a "wound" in a cell monolayer. Cells were seeded in a culture plate to reach > 90% confluence. The monolayer was gently scratched using a sterile pipette tip, generating a uniform linear gap. Cells were washed with PBS to remove detached cells and debris. Medium was replaced with low-serum (1–2% FBS) medium to minimize proliferation interference. Images of the scratch were captured at 0 h (baseline) and at 48-h intervals using phase-contrast microscopy. Consistent culture conditions (temperature, CO₂) were maintained. Migration was quantified by measuring the scratch width over time using image analysis software (ImageJ). Migration rate was calculated as: [(Initial scratch area − Final scratch area) / Initial scratch area] × 100%.

### Statistical analysis

In this study, statistical analyses were performed using Student's t-test for normally distributed data or Wilcoxon rank-sum test for nonparametric data. For high-dimensional analyses involving multiple hypothesis testing, FDR correction was systematically applied using the Benjamini–Hochberg procedure (FDR < 0.05) to control Type I error. For functional assays, data are presented as mean ± SD from three independent experiments. Survival data were analyzed via Kaplan–Meier curves with log-rank test. Multivariate Cox regression included the assessment of proportional hazards assumptions using Schoenfeld residuals. All analyses were conducted using GraphPad Prism or R, with significance levels denoted as *p < 0.05, **p < 0.01, ***p < 0.001, and ****p < 0.0001.

## Results

### Characterization of tumor tissue composition and gene expression profiles in CRC patients and healthy individuals

**Fig. 1:**
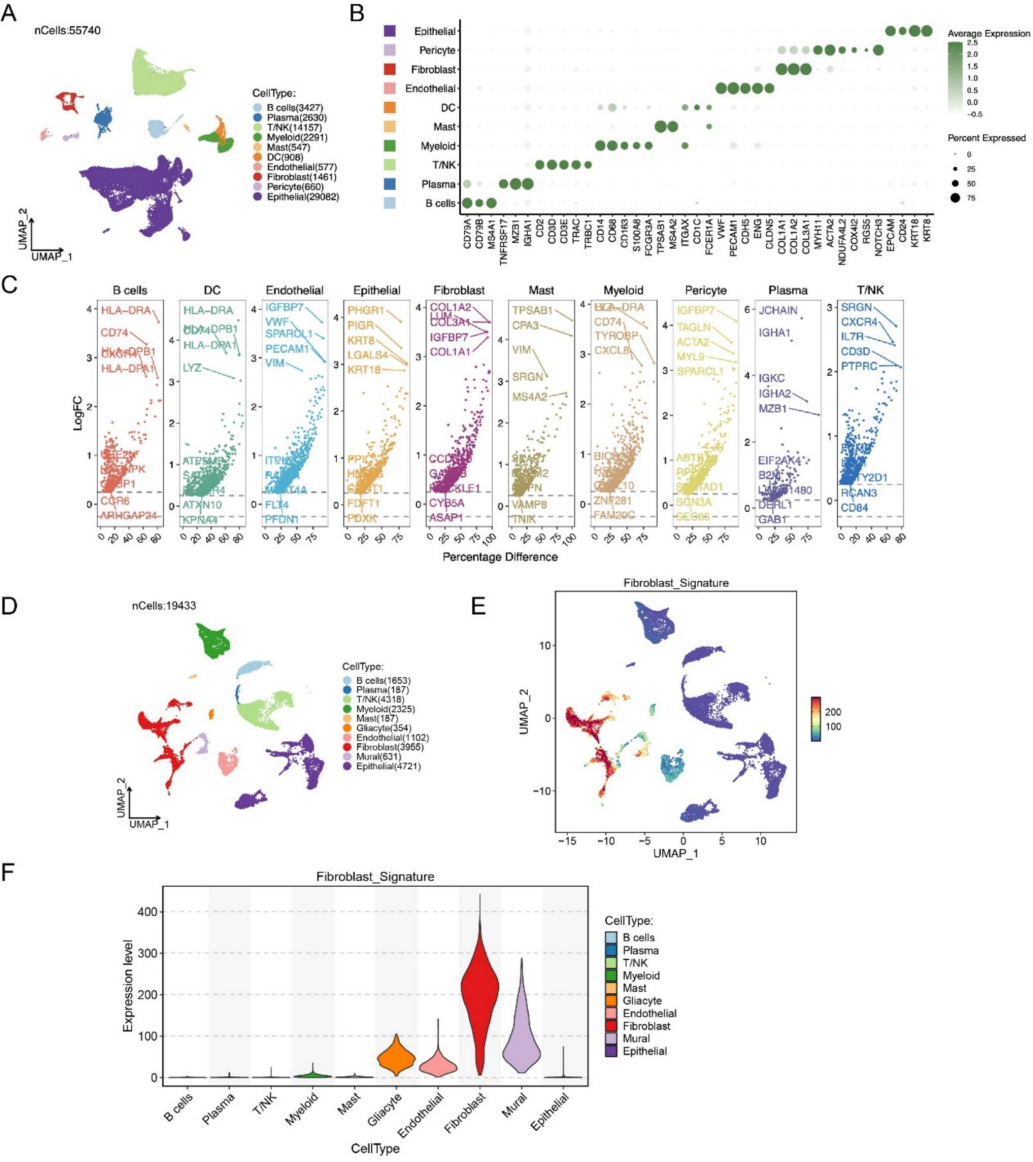
Overview of single-cell analysis. **A** The UMAP plot comprising 55,740 single cells in the GSE166555 dataset. **B** Dot plot showing the expression of marker genes for ten cell types. **C** Differentially expressed genes (DEGs) across the ten cell types between CRC patients and controls. **D** The UMAP plot comprising 19,433 single cells in the GSE144735 dataset. **E** UMAP visualization showing fibroblast signature score distribution across all cell types. **F** Violin plot showing the fibroblast signature expression levels of different cell types

To identify cell subtype-specific genes in CRC tumor tissues, we performed single-cell transcriptomic analysis on the GSE166555 dataset using the Seurat package in R. Following a standardized pipeline, 55,740 cells were obtained by integrating the expression patterns of tumor tissues. Cell clusters were annotated based on canonical marker genes and the COSG method, ultimately identifying 10 distinct cellular subpopulations as illustrated in UMAP plot (Fig. 1A). These ten cell types included: B cells, plasma cells, T/NK cells, myeloid cells, DC cells, mast cells, epithelial cells, endothelial cells, pericyte cells, and fibroblast cells (Fig. 1A, B). DEGs within these cell subtypes were displayed in the volcano plot (Fig. 1C). Among them, differential gene analysis revealed 99 CAF-specific genes between CRC patients and healthy individuals, including both up- and downregulated genes (Fig. 1C) (FDR < 0.05).

To validate the results, we incorporated an independent validation cohort from the GSE144735 dataset (Fig. 1D). UMAP visualization revealed that fibroblast populations were effectively captured consistently with the discovery cohort (Fig. 1D, E). The violin plot demonstrates that fibroblast signature expression is predominantly elevated in fibroblast cells compared to other cell types (B cells, plasma, T/NK, myeloid, mast, endothelial, and epithelial cells) (Fig. 1F), indicating high specificity of fibroblast signature.

### Consensus clustering according to the CAF-specific genes

**Fig. 2:**
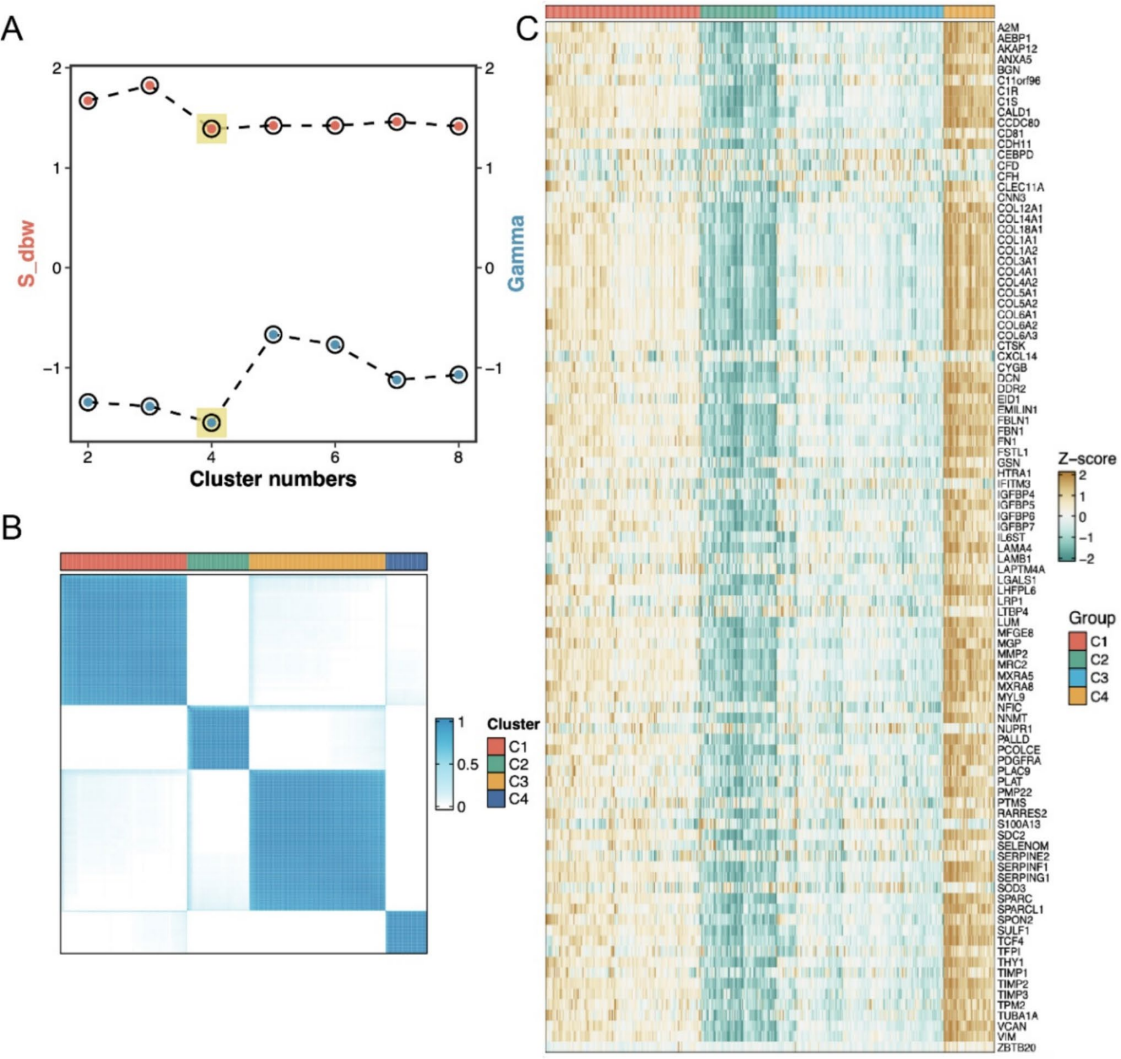
The result of consensus clustering according to the 99 CAF-specific genes for the CRC patients. **A** Optimal cluster number was determined as k = 4 through the consensus clustering algorithm. **B** Heatmap illustrating expression patterns of CAF-specific genes across these four subtypes. **C** Hierarchically clustered heatmap depicting expression levels of CAF-specific genes in four subtypes (C1–C4)

Given the critical role of CAFs in tumorigenesis and progression, we performed consensus clustering analysis on CRC tumor samples based on CAF-specific genes using the consensusClusterPlus package. The optimal cluster number was determined as k = 4 through the consensus clustering algorithm, with a heatmap illustrating four distinct CAF subtypes (C1–C4) (Fig. 2A, B). Comparative analysis of CAF-specific gene expression across the four subtypes revealed distinct molecular profiles (Fig. 2C). Notably, C4 showed significantly elevated expression of the majority of CAF-specific genes compared to other subtypes. This transcriptional prominence suggests that C4 may represent a functionally distinct CAF subpopulation with enhanced fibroblast-associated signatures, potentially driving specific biological processes or cellular fate determination in the CRC microenvironment.

### Stromal score and functional enrichment analysis

**Fig. 3:**
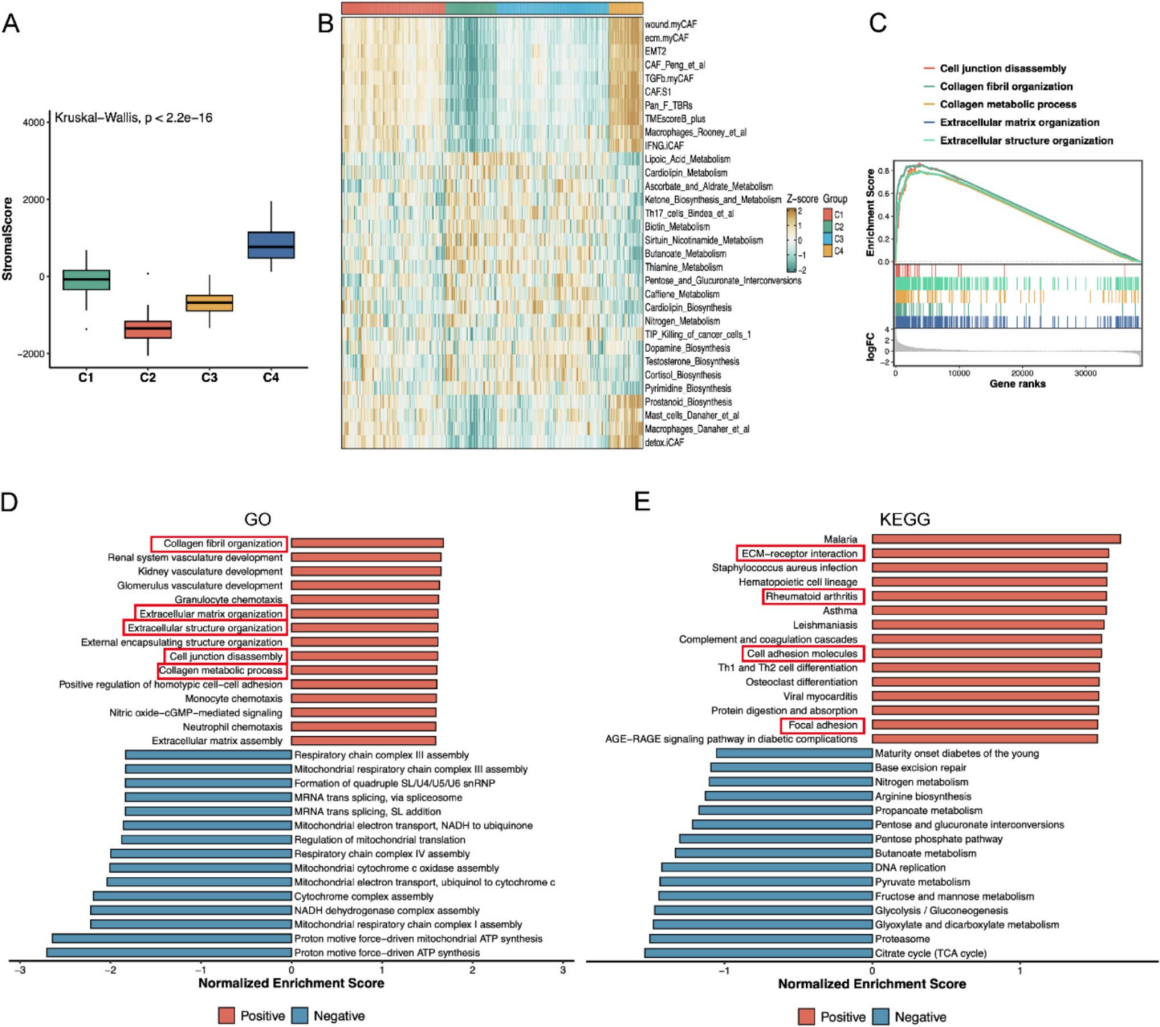
Stromal score and functional enrichment analysis. **A**. The stromal scores in C1–C4 subtypes. **B** Heatmap of signature score quantification in C1–C4 subtypes. **C** GSEA analysis of the C4 subtype. **D**, **E** GO and KEGG enrichment analysis of the C4 subtype

Given the obvious association between CAFs and stroma, we analyzed the stromal scores for the four subtypes. ESTIMATE algorithm analysis revealed significantly elevated stromal scores in the C4 subtype compared to other subtypes (Kruskal–Wallis test) (Fig. 3A). Subsequent signature score quantification using the IOBR package demonstrated predominant enrichment of stromal activation signatures (e.g., wound. myCAF, eco. myCAF, TGFβ. myCAF, EMT2, and CAF.S1) in C4 subtype (Fig. 3B). As shown in Supplementary Fig. 1, GO enrichment results of C1, C2, and C3 represent a developmental matrix organization subtype, metabolic and energy production subtype, and innate immunity and host defense subtype, respectively. In contrast to these subtypes, C4 was predominantly enriched in ECM-related pathways in GSEA analysis (Fig. 3C). Consistently, GO enrichment analysis characterized C4 as a pathological ECM remodeling subtype (Fig. 3D), while KEGG pathway analysis further demonstrated enrichment in "ECM-receptor interaction," "cell adhesion molecules," and "focal adhesion" (Fig. 3E). These enrichment patterns identify C4 as a hyperactivated stromal subtype orchestrating tumor-promoting microenvironmental remodeling.

### Prognostic value of CAF-specific genes in CRC

**Fig. 4:**
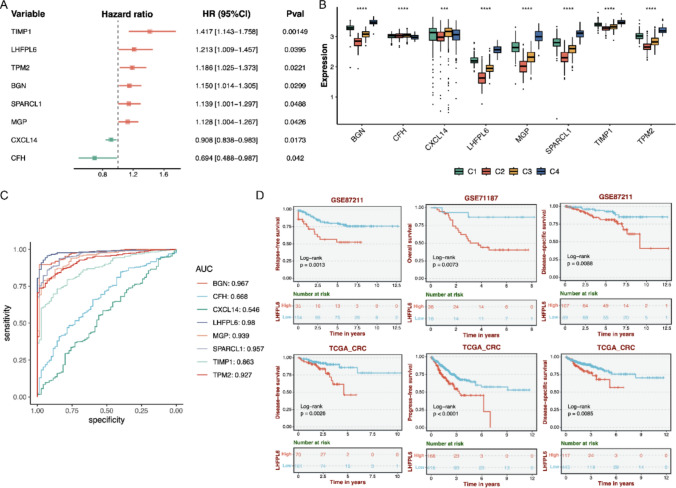
Identification and validation of candidate genes to predict the prognosis of CRC. **A** The results of univariate Cox regression analysis for the 99 CAF-specific genes utilizing the data of TCGA CRC. **B** The expression levels of eight candidate genes in four clusters. **C** The AUC values of eight candidate genes for C4 subtype classification. **D** Survival analysis for LHFPL6 in different datasets

To assess the prognostic value of CAF-specific genes in CRC, univariate Cox regression analysis was performed for the 99 CAF-specific genes. The analysis identified eight prognosis-associated genes, comprising six risk genes (HR > 1, *p* < 0.05) and two protective genes (HR < 1, *p* < 0.05) (Fig. 4A). Kaplan–Meier survival analysis in the TCGA-CRC cohort further confirmed significant stratification of overall survival for all eight genes (log-rank *p* < 0.05) (Supplement. Figure 2). Subtype-specific expression profiling demonstrated the differentially expressed levels of the eight CAF-specific genes among the four clusters (Fig. 4B). ROC analysis revealed differential discriminative abilities across the 8-gene panel for C4 subtype classification, with LHFPL6 exhibiting the highest specificity and sensitivity (AUC = 0.98) (Fig. 4C).

In addition, multi-cohort validation confirmed that elevated LHFPL6 expression was correlated with reduced recurrence-free survival (RFS: *p* = 0.0013, GSE87211), disease-specific survival (DSS: *p* = 0.0088, GSE87211), overall survival (OS: *p* = 0.0073, GSE71187). In TCGA-CRC cohort, high LHFPL6 expression was significantly associated with worse disease-free survival (DFS: *p* = 0.0026), progress-free survival (PFS: *p* < 0.0001), and DSS (*p* = 0.0085) (Fig. 4D). Finally, multivariate Cox regression analysis identified that LHFPL6 is an independent prognostic factor for CRC (HR = 5.889, 95% CI: 1.367–25.364, *p* = 0.017), after adjusting for age, gender, and tumor stage (Table 1). Patients with high LHFPL6 expression had nearly sixfold increased risk of adverse outcomes compared to those with low expression, demonstrating the strongest prognostic impact among all analyzed clinicopathological variables.

**Table 1:** The results of multivariate Cox regression analysis

Variable	Coefficient	Hazard ratio	95% CI Lower	95% CI Upper	Z-value	*P* value
LHFPL6	1.773	5.889	1.367	25.364	2.38	0.017
Age	0.619	1.857	0.674	5.115	1.198	0.231
Gender	−0.079	0.924	0.377	2.265	−0.172	0.863
Stage	0.95	2.587	0.9	7.435	1.764	0.078

### Association of LHFPL6 with CAF score and identification of co-expression network

**Fig. 5:**
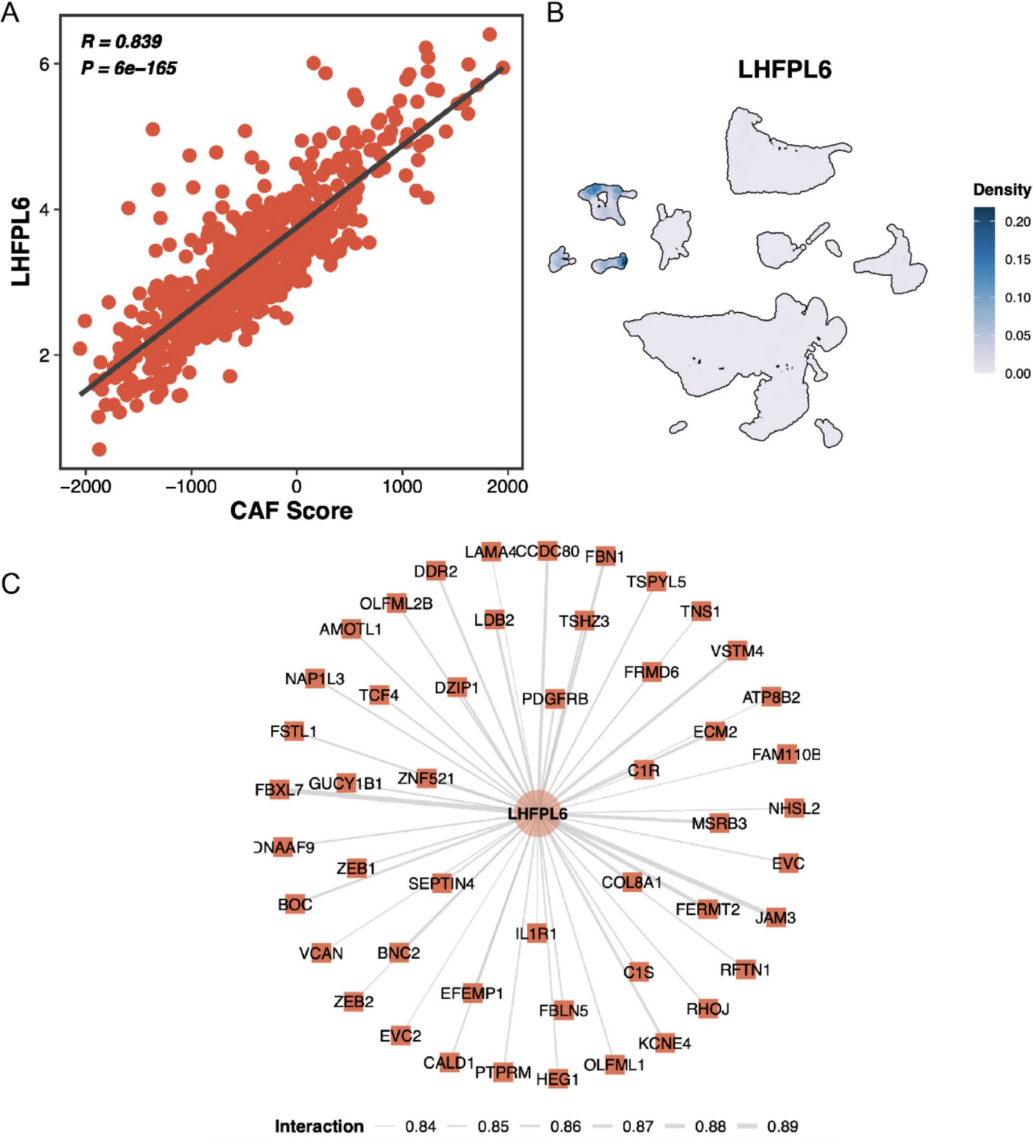
The function and localization of LHFPL6. **A** Association of LHFPL6 with CAF score. **B** Localization analysis of LHFPL6 in cell subpopulations. **C** Co-expression gene network analysis of LHFPL6

To clarify the correlation between LHFPL6 and CAF scores, we performed correlation analysis in the TCGA-CRC cohort, which revealed a significant positive association between LHFPL6 expression and CAF scores (Fig. 5A). Subpopulation localization analysis further demonstrated that LHFPL6 was predominantly expressed in fibroblast subpopulations (Fig. 5B). These results reinforce the role of LHFPL6 as a CAF-specific gene molecular feature for prognostic prediction in CRC. To explore potential functional associations of LHFPL6 in CRC, we performed co-expression network analysis using the TCGA-CRC cohort. The top 50 genes showing correlated expression patterns with LHFPL6 were identified based on Pearson correlation coefficients (|r|> 0.6, FDR < 0.05) (Fig. 5C). These co-expressed genes may provide insights into biological processes in which LHFPL6 potentially participates.

### Pathway enrichment analysis about the co-expressed genes of LHFPL6

**Fig. 6:**
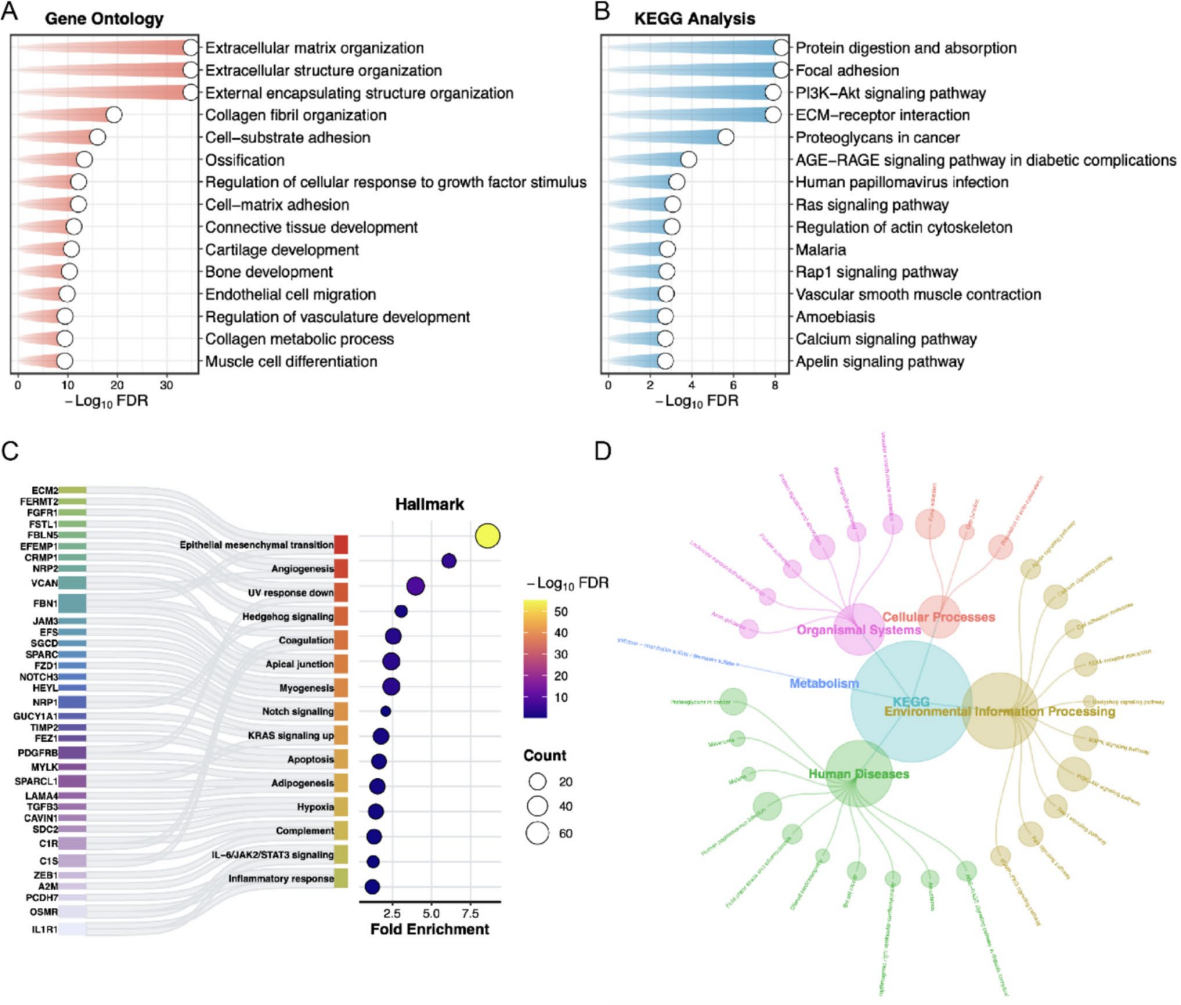
Enrichment analysis of LHFPL6-co-expressed genes. **A**, **B** The results of GO/KEGG pathway enrichment analysis for co-expression genes of LHFPL6. **C** The results of Hallmark analysis for co-expression genes of LHFPL6. **D** KEGG pathway network of LHFPL6-co-expressed genes

To further elucidate the role of LHFPL6 in tumorigenesis and progression, we performed GSEA functional enrichment analysis on LHFPL6-co-expressed genes using ClusterProfiler. The results revealed that LHFPL6 expression correlates with gene signatures involved in stromal remodeling, cell adhesion, and epithelial–mesenchymal transition (EMT) (Fig. 6). GO enrichment analysis identified significantly enriched biological processes including ECM organization and extracellular structure organization and so on (Fig. 6A). KEGG pathway enrichment analysis demonstrated prominent enrichment in pathways such as protein digestion and absorption and focal adhesion and so on (Fig. 6B). Hallmark gene set analysis further confirmed that LHFPL6-co-expressed genes were enriched in critical pathways, where ECM2, FERMT2, and FBN1 were identified as signature genes associated with the EMT pathway, while FGFR1, FSTL1, and VCAN emerged as characteristic genes involved in the angiogenesis pathway (Fig. 6C). Furthermore, network plot shows KEGG pathway network of LHFPL6-co-expressed genes, encompassing cellular processes, metabolism, organismal systems, human diseases and environmental information processing (Fig. 6D). These findings suggest that LHFPL6, as a CAF-specific gene, may participate in tumorigenesis and progression through mechanisms associated with tumor microenvironment remodeling. Basic in vitro investigations were subsequently performed to further characterize the functional mechanisms of the gene.

### LHFPL6 drives CRC progression through CAF-tumor crosstalk

**Fig. 7:**
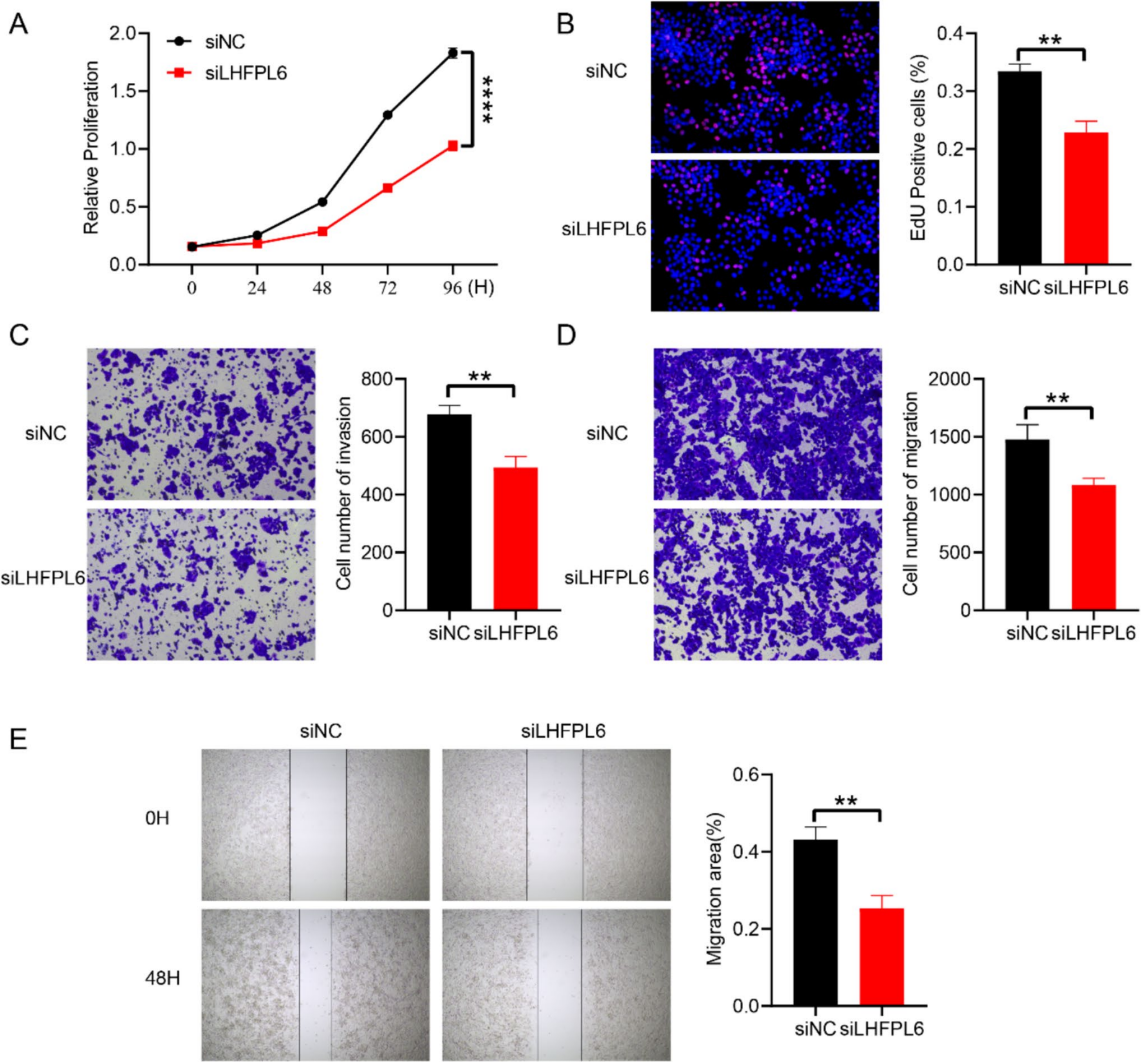
LHFPL6 knockdown in CAFs attenuates SW480 malignant phenotypes. All functional assays were performed following siRNA-mediated LHFPL6 knockdown in CAFs and subsequent 48-h co-culture with SW480 adenocarcinoma cells. **A** CCK-8 proliferation kinetics showing significant viability reduction. **B** EdU incorporation assay quantifying S-phase suppression. **C** Matrigel invasion capacity assessed by Transwell chambers. **D** Migration potential through uncoated Transwell membranes. **E** Scratch wound closure dynamics monitored by time-lapse imaging. Data represent mean ± SEM from three independent biological replicates (n = 3 technical replicates/group); **p < 0.01, ****p < 0.0001 by two-tailed Student's t-test

To delineate the regulatory role of LHFPL6 in CRC carcinogenesis, we engineered LHFPL6-knockdown CAFs using sequence-validated siRNA duplexes (siLHFPL6) with scrambled siRNA (siNC) as isogenic controls. These genetically modified CAFs were co-cultured with SW480 adenocarcinoma cells in a Transwell system (0.4 μm pore, Corning) for 48 h to investigate CAF-tumor crosstalk. Functional experiments demonstrated that LHFPL6 silencing in CAFs significantly attenuated SW480 malignant phenotypes: CCK-8 proliferation assays revealed marked viability reduction (44.4% inhibition vs. controls) (Fig. 7A), corroborated by EdU-based S-phase suppression (30.1% decrease) (Fig. 7B). Transwell invasion and migration analyses showed 27.1% and 26.7% impairment in SW480 motility and invasiveness, respectively (Fig. 7C, D), while scratch wound healing assays confirmed delayed closure kinetics (siNC vs siLHFPL6, 43.3% vs. 25.4% at 48 h) (Fig. 7E). These findings collectively demonstrate that LHFPL6 knockdown in CAFs remodels the tumor-permissive niche into a growth-restrictive microenvironment, establishing LHFPL6 as a CAF-specific prognostic biomarker and potential therapeutic target in CRC.

## Discussion

CRC continues to pose substantial clinical challenges worldwide, largely attributable to the complex interactions within the TME that fundamentally govern tumor progression and therapy resistance [20, 21]. As a dominant stromal element in solid tumor microenvironments, CAFs exhibit substantial functional heterogeneity and phenotypic plasticity. There is growing scientific focus on disrupting the crosstalk between CAFs and malignant cells, an emerging therapeutic approach to impede cancer carcinogenesis and optimize therapeutic efficacy in patients [22]. Not only in solid tumors, but also in hematologic malignancies such as multiple myeloma, CAFs, ECM remodeling, and their crosstalk with tumor cells represent central components of the tumor microenvironment [23]. For instance, bone marrow stromal cells can adopt CAF phenotypes, secreting various cytokines to stimulate tumor cell growth, infiltration, and endosteal niche reconstruction [24]. In this study, we focused on the identification of CAF subtypes based on CAF-specific genes and highlighted the previously unexplored role of LHFPL6 in prognostic prediction and potential molecular mechanisms involved in CRC progression. Beyond CRC, these mechanistic insights into how LHFPL6 in CAFs modulates tumor behavior may have broader implications for understanding CAF biology in diverse malignancies.

ScRNA-seq uncovers cellular heterogeneity at single-cell resolution, allowing precise characterization of diverse cellular subpopulations and discovery of significant subsets that exert pivotal influences on oncogenesis and malignant evolution [25]. The present study capitalizes on scRNA-seq to uncover the alterations in cellular heterogeneity of CRC, identifying 99 CAF-specific genes. Based on these CAF-specific genes, we further identified four CAF subtypes. Notably, in the C4 subtype, these genes were significantly highly expressed, indicating that the C4 subtype is closely related to the CAF characteristics represented by CAF-specific genes in terms of biological function or cell fate. CAFs can alter ECM composition and significantly influence tissue stiffness, a phenomenon known as matrix rigidity [26]; therefore, we evaluated the stromal scores and found that the C4 subtype was mainly related to matrix activation. Elevated matrix rigidity is mechanistically associated with enhanced tumor cell invasion and migration across multiple malignancies [27]. Consistently, GSEA enrichment analysis showed that the genes in the C4 subtype were enriched in ECM-related processes, such as ECM organization, extracellular structure organization, and EMC–receptor interaction. The aforementioned results indicate that the C4 subtype may promote cancer progression by regulating matrix rigidity in CRC.

The univariate regression results indicated that only eight genes (BGN, CFH, CXCL14, LHFPL6, MGP, SPARCL1, TIMP1, TPM2) could serve as prognostic indicators for CRC. Kaplan–Meier analysis further confirmed these genes were associated with survival outcomes of the patients. Furthermore, we revealed the expression levels of these genes in the four cell subtypes, and the ROC analysis for C4 subtype prediction showed that LHFPL6 had the highest sensitivity, highlighting its potential as a key player in the C4 subtype. In addition, multivariate Cox regression analysis identified that LHFPL6 is an independent prognostic factor for CRC, after adjusting for age, gender, and tumor stage. LHFPL6 (tetraspanin subfamily member 6), localized to chromosome 13q, is a member of the lipoma HMGIC fusion partner gene family, which was reported as a translocation partner of the high mobility group A2 (HMGA2) gene [28, 29]. HMGA2-mediated transcriptional regulation is clinically associated with dysregulated cancer progression, accelerated metastasis, and adverse overall survival in multiple malignancies, including CRC [30, 31]. Therefore, it is possible that LHFPL6 could be involved in the progression of CRC. Here, we revealed, for the first time, that high expression of LHFPL6 was linked to poor outcomes in multiple CRC cohorts. LHFPL6 also served as a risk factor for unfavorable survival in gastric cancer [32], which was consistent with our study. In addition to its prognostic role, we also provided evidence that LHFPL6 was mainly expressed in CAF subsets and significantly correlated with the CAF score. The results of functional enrichment showed that LHFPL6 expression correlated with matrix remodeling, cell adhesion, and EMT. During EMT, epithelial cells undergo structural alterations that disrupt intercellular junctions and reorganize ECM-anchored adhesion complexes, thereby facilitating ECM remodeling [33]. The collaborative interplay between EMT and ECM influences the metastatic process; this directly enhances the stability and deposition of collagen within the ECM of metastatic tumor tissues, which is a direct result of the amplification of collagen gene expression [34]. These findings highlight LHFPL6's potential as a key player in regulating tumorigenesis. Thus, we further engineered LHFPL6-knockdown CAFs and co-cultured them with SW480 adenocarcinoma cells in a Transwell system. We found that LHFPL6 silencing in CAFs significantly attenuated SW480 tumor proliferation, invasion, and migration. This may be because LHFPL6 ablation in CAFs remodels the tumor-permissive niche into a growth-restrictive microenvironment, suggesting a potential molecular mechanism involved in regulating tumorigenesis.

This study has several limitations. First, our functional experiments were conducted solely with SW480 cells, and validation across multiple cell lines is needed. Second, the molecular mechanisms by which LHFPL6 regulates ECM remodeling and EMT signaling require deeper investigation through animal models and expanded cellular assays. Third, although LHFPL6 shows promise as a potential therapeutic target, CAF-targeted therapies have encountered translational barriers, including tumor heterogeneity and variable treatment responses. Future work incorporating spatial transcriptomics and comprehensive preclinical and clinical validation will help to fully understand CAF biology and develop effective therapeutic strategies.

In conclusion, this study performed an integrative analysis of CAF cell subpopulations in CRC and demonstrated that LHFPL6 is associated with CAF-mediated tumor progression, establishing LHFPL6 as a promising prognostic biomarker and potential therapeutic target for CRC.

## Supplementary Information

Below is the link to the electronic supplementary material.

Supplementary file1 (TIF 17624 KB)The Supplementary files should be renumbered to match their order of appearance in the text, where we have changed to the right number. Please change the current "Supplementary file 2" to "Supplementary file 1", current "Supplementary file 1" to "Supplementary file 2".Thank you.

Supplementary file2 (TIFF 2166 KB)

## Data Availability

The data of this study are available from the corresponding author upon reasonable request.
