# Supplementary figures and images for: Integrative analysis identifies LHFPL6 as a CAF-specific prognostic biomarker in colorectal cancer
Source: Clin Exp Med. 2025 Nov 18;26(1):23. doi: 10.1007/s10238-025-01954-y (PMC12628457; doi:10.1007/s10238-025-01954-y)

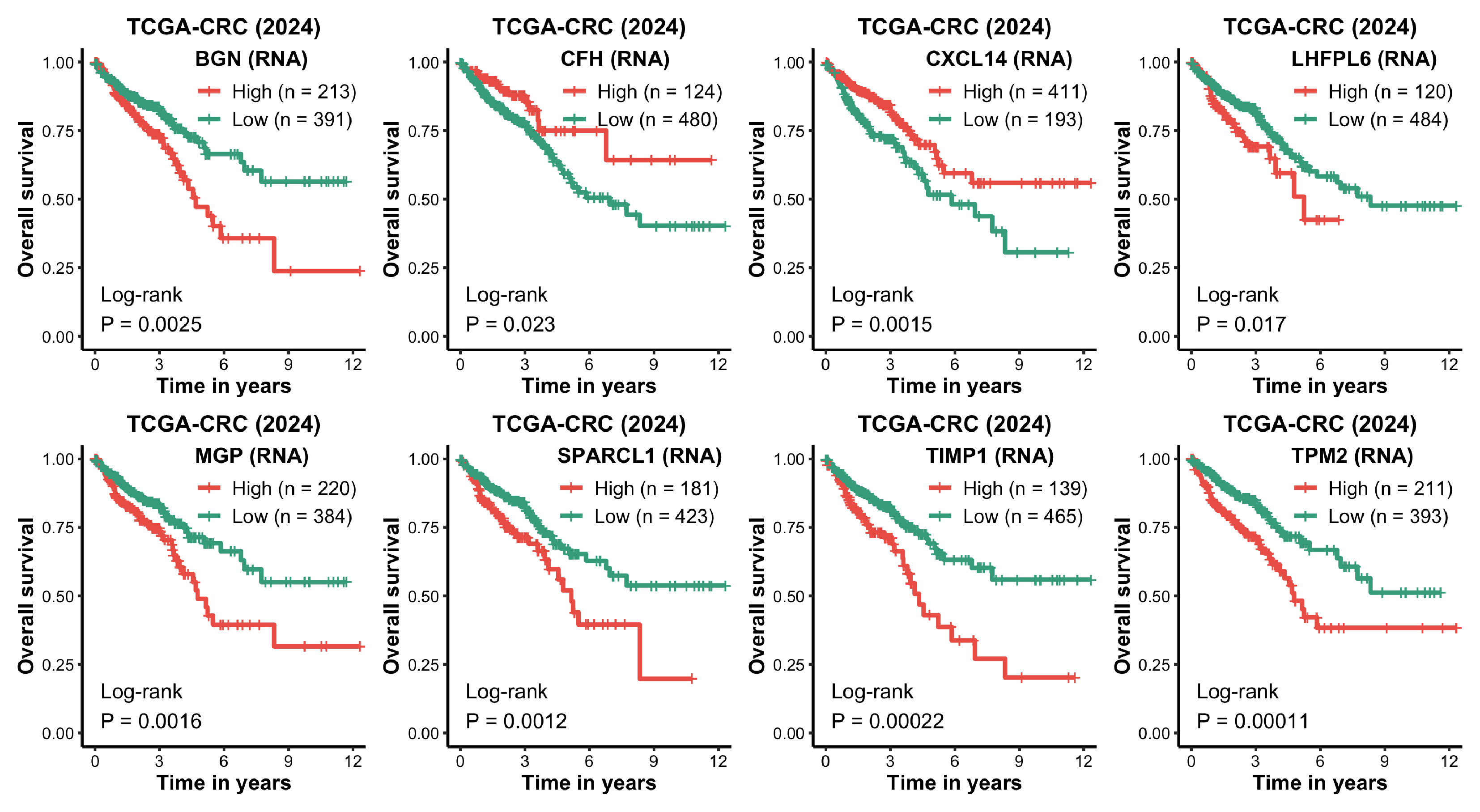

Supplement: Supplementary file 1 — Supplementary file1 (TIF 17624 KB)The Supplementary files should be renumbered to match their order of appearance in the text, where we have changed to the right number. Please change the current "Supplementary file 2" to "Supplementary file 1", current "Supplementary file 1" to "Supplementary file 2".Thank you. [file 10238_2025_1954_MOESM1_ESM.tif]

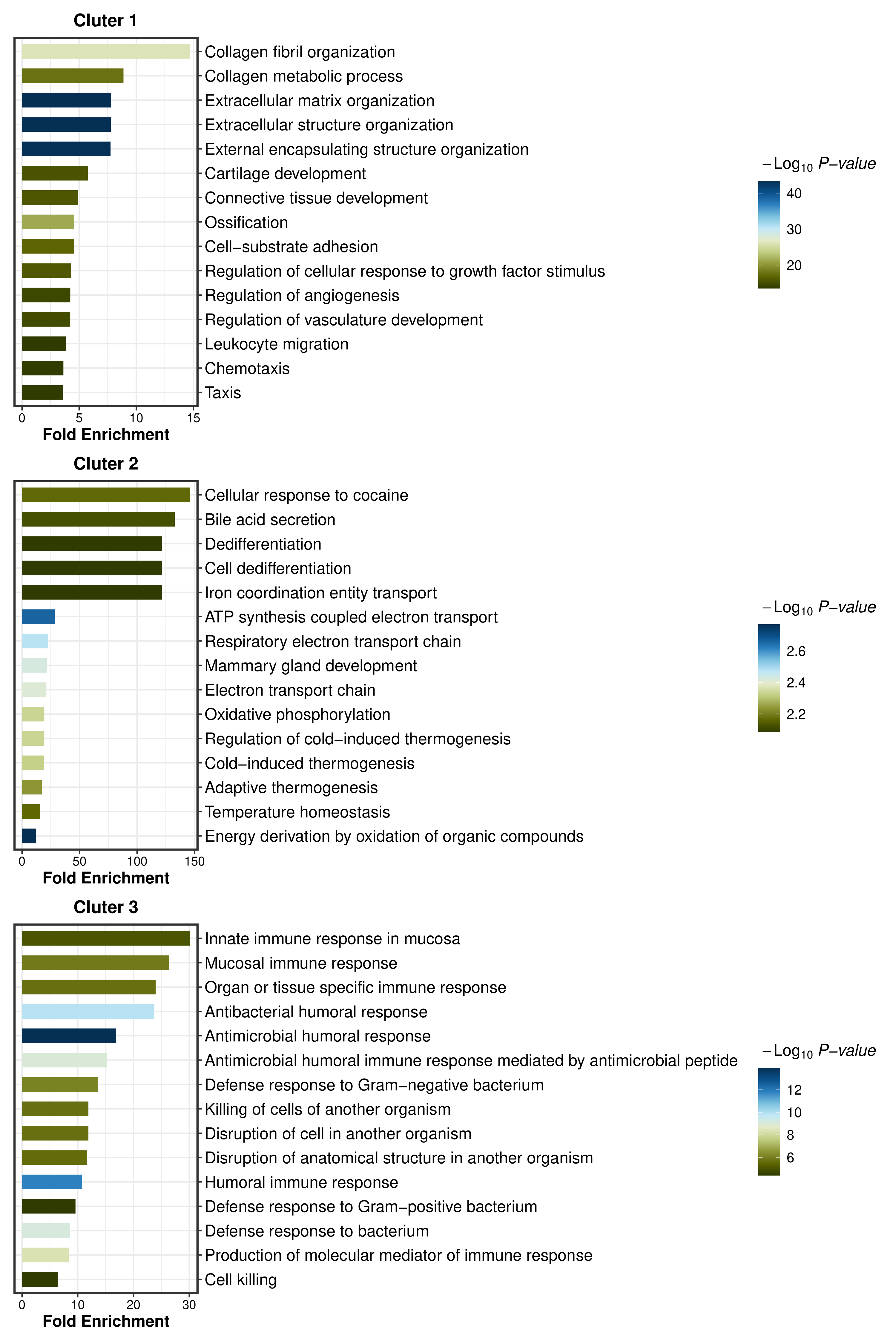

Supplement: Supplementary file 2 — Supplementary file2 (TIFF 2166 KB) [file 10238_2025_1954_MOESM2_ESM.tiff]
